# Stay Active, Stay Healthy: A Cross-Sectional View of the Impact of Physical Activity Levels on Health Parameters of Older Adults Institutionalized in Nursing Homes of Barcelona

**DOI:** 10.3390/life15030412

**Published:** 2025-03-06

**Authors:** Sergi Rodríguez-Rodríguez, Guillermo R. Oviedo, Carlos López-de-Celis, Joan Bosch-Sabater, Esther Jovell-Fernández, Albert Pérez-Bellmunt, Leonor Cuadra-Llopart, Jacobo Rodríguez-Sanz

**Affiliations:** 1 Department of Medicine, Faculty of Medicine and Health Sciences, Universitat Internacional de Catalunya, 08195 Barcelona, Spain; srodriguezr@uic.es (S.R.-R.); jboschs@uic.es (J.B.-S.); ejovell@cst.cat (E.J.-F.); aperez@uic.es (A.P.-B.); jrodriguezs@uic.es (J.R.-S.); 2 Actium Functional Anatomy Research Group, Sant Cugat del Vallès, 08195 Barcelona, Spain; carlesldc@uic.es; 3 Department of Kinesiology, Mississippi State University, Starkville, MS 38677, USA; gro16@msstate.edu; 4 Fundació Institut Universitari per a la Recerca a l’Atenció Primària de Salut Jordi Gol i Gurina (IDIAPJGol), 08007 Barcelona, Spain; 5 Department of Epidemiology, Consorci Sanitari de Terrassa, 08227 Terrassa, Spain; 6 Department of Geriatric Medicine, Consorci Sanitari de Terrassa, 08227 Terrassa, Spain

**Keywords:** aging, older adults, institutionalization, sarcopenia, sedentarism, physical activity

## Abstract

(1) Background: Institutionalized older adults represent a vulnerable population. It is important to understand that higher levels of physical activity in older adults are associated with less risk of cardiovascular diseases, better cognition, and lower inflammaging and sarcopenia levels. The main objective was to evaluate the differences in health parameters in institutionalized older adults who perform different levels of weekly physical activity. The secondary objective was to analyze if weekly physical activity levels are a predictor of health parameters. (2) Methods: A cross-sectional observational study was conducted in nursing homes with adults over 75 years of age. A total of 76 participants was divided into three groups based on their weekly physical exercise frequency (1 day/week, 2 days/week, and 3 days/week). We measured demographic and anthropometric variables, along with cognitive level through the Mini Exam of Lobo. Handgrip strength, leg muscle strength, and power were also evaluated, and C-reactive protein levels were assessed through blood tests. Physical performance was measured using the Short Physical Performance Battery and walking speed. (3) Results: Significant differences were found in body mass index (*p* < 0.01; ES = 0.96), muscular strength (*p* < 0.01; ES = 0.70), and power (*p* < 0.01; ES = 1.09), Short Physical Performance Battery (*p* < 0.01; ES = 1.46) and walking speed (*p* < 0.01; ES = 0.87), cognitive function (*p* < 0.01; ES = 1.21), and C-reactive protein levels (*p* < 0.01; ES = 1.73), favoring the group who performed 3 days/week of physical activity. (4) Conclusions: Institutionalized older adults with three days per week of physical activity have greater physical and muscle function and less cognitive decline. Three days of weekly physical activity is associated with systemic inflammation and better cognitive status in institutionalized older adults.

## 1. Introduction

Significant advances have been made in understanding the pathways involved in aging, which is recognized as a complex set of biological processes [1]. We consider aging to encompass sub-organismal biological processes leading to declines in function with the passing time, including hierarchical scales ranging from molecular to clinical manifestations, like decreased muscle strength and physical performance [2,3]. This process is therefore a multifactorial phenomenon, requiring a holistic perspective to fully capture the intricate interplay between biological, environmental, and social factors that shape functional aging [4].

**Figure 1:**
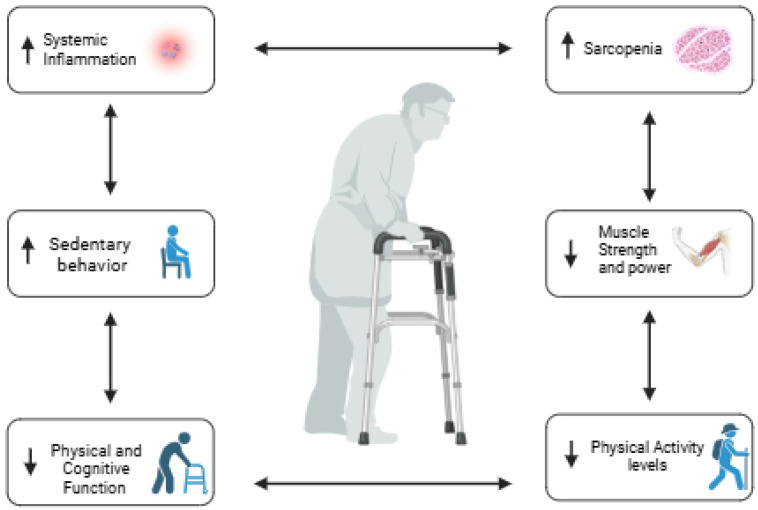
Interplay between functional decline, systemic inflammation, frailty, and physical activity. Created in BioRender.com (accessed on 4 March 2025).

To counteract these changes, geriatric assessment and multifactorial interventions have been widely recommended [5,6]. Among these, physical activity (PA) emerges as a cornerstone strategy, with well-documented benefits for mitigating age-related decline and promoting healthy aging [5]. However, the effectiveness of such recommendations often depends on individual circumstances, including social support, prior sedentary habits, and physical or cognitive limitations [7,8]. Institutionalized older adults, in particular, represent a vulnerable population characterized by lower levels of physical activity [9,10], higher prevalence of chronic conditions (e.g., diabetes, hypertension), and pronounced health challenges such as inflammaging—a systemic, chronic low-grade inflammation associated with aging that exacerbates functional decline, which is thought to be the long-term result of chronic physiological stimulation of the innate immune system, mainly caused by the dysregulation of the cellular receptors of self and non-self, increasing several inflammatory cytokines such as interleukin (IL)-1, IL-6, and tumor necrosis factor-alpha [11,12]. However, a systematic review reveals that only 60% of the residents institutionalized receive physiotherapy services [13]. This population often experiences a vicious cycle, where sedentary behavior accelerates physical deterioration, including the loss of strength, impaired balance, and reduced mobility, increasing the risk of (pre) sarcopenia, which in turn perpetuates inactivity and further fuels inflammaging (Figure 1) [12,14]. This is possible because institutionalized older people have worse health status indicators, with a high prevalence of high inflammatory stress. These patients showed comorbidities such as hypertension, diabetes, and kidney disease, and very high levels of systemic inflammatory biomarkers [10], reducing the regular practice of physical exercise.

Even so, it is important to emphasize that the more often a person is physically active, the better their physical capability [15]. In middle and old age, higher levels of physical activity and fitness are associated with lower mortality risk, better health, and longevity in old age, even for those who start exercising later in life. Regular physical activity reduces the risk of cardiovascular and metabolic diseases, improves cognitive function, and minimizes inflammaging development and the risk of sarcopenia [15,16]

The fact is that an increasing proportion of older adults reside in long-term residential care facilities, where they typically spend most of their time sitting or lying down, and rarely perform physical activity (once per week or less in most cases), which potentially accelerates their physical decline [17]. Although a heterogeneous population group, older adults in residential care commonly undergo rapid impairment of physical function and more than half lose the ability to independently perform at least one activity of daily living within the first 2 years of admission to a long-term care facility [17]. This is why understanding the specific impact of physical activity on health parameters in this specific population is essential to developing targeted interventions that break this cycle and support healthier aging outcomes.

To date, different studies have studied the post-effects of different physical activity interventions in institutionalized older adults on health parameters [17,18,19], most of these studies have employed experimental or longitudinal designs, focusing on specific interventions and their effects over time, but no cross-sectional studies have been conducted evaluating the differences in health parameters of institutionalized older adults > 75 years old based on the number of days of weekly physical activity. Such an approach could provide a more realistic picture of the impact of physical activity under everyday conditions, helping to identify trends and associations that could inform future health promotion strategies in long-term care facilities. For this reason, the main objective of the study is to evaluate the differences in health parameters in institutionalized older adults who perform different levels of weekly physical activity, and secondarily, to analyze if weekly physical activity levels are a predictor of health parameters.

## 2. Materials and Methods

### 2.1. Study Design

A cross-sectional observational study was conducted in nursing homes with institutionalized older adults over 75 years of age between December 2022 and December 2023. The ethical principles for research on human beings established in the Declaration of Helsinki, last revised in Fortaleza in October 2013, have been respected. The study was approved by the Research Ethics Committee of the Consorci Sanitari de Terrassa (01-22-175-053) and the STROBE Statement was followed. The sample size for the study was calculated with the GRANMO program, considering the data from the Ministry of Social Rights and “Agenda 2030” on adults over 75 years of age institutionalized in nursing homes in Spain. A confidence level of 0.95 was applied, an estimate of the population proportion of 0.4, and a precision of the estimate for the confidence level of 0.11. A loss to follow-up rate of 0% was estimated considering that it is a cross-sectional study. The POISSON approximation was used. A total sampling of 76 older adults was collected in our study.

All participants included in the study previously gave us written informed consent to participate in the study and publish the data. In those participants with mild/moderate cognitive impairment, consent was obtained from their dependent relatives.

### 2.2. Participants

The inclusion criteria were (a) persons over 75 years of age (b) institutionalized in a nursing home and (c) performing between 1 and 3 days of weekly physical activity at the nursing home (PA). Exclusion criteria were (a) previous bone fracture in the last 6 months, (c) uncontrolled symptomatic cardiovascular or respiratory disease, (d) current cancer under treatment, or (e) inability to understand the information provided by the researchers.

The participants included in the study were divided into three groups based on the days a week they perform PA (1 day/week, 2 days/week, or 3 days/week) within the residences where they lived. No more groups of days were held in these residences since, due to therapist logistics, the minimum number of weekly days of physical activity was 1 and the maximum was 3 days/week. In the residences where we went to collect the data, the weekly PA levels performed by the elderly were calculated by the staff of the residence through the medical history they present upon admission, the objectives of the family members, and the initial evaluation of the physiotherapist through joint mobility in the upper and lower extremities, 5-Sit To Stand (5-STS), and Barthel scale scores (0–100). Prior to the start of the study, a control session was conducted with physiotherapists and assessment staff at each residence. This session included detailed instructions on the administration and scoring of the Barthel Index and the 5-STS test, ensuring a uniform understanding and realization of the procedures. We ensured that the Barthel Index from Mahoney et al. [20] was used. This scale has good inter-observer reliability, with Kappa indices (0.47–1.00), and intra-observer reliability with Kappa indices (0.84–0.972324). Regarding the evaluation of internal consistency, a Cronbach’s alpha (0.86–0.92) was observed for the original version [21]. Institutionalized older adults scoring below 61 on the Barthel Index received 1 day of activity per week; those scoring 61–90 received 2 days; and those scoring 90–100 received 3 days per week. This division was previously agreed upon by the therapists based on the criteria of Shah et al. [22] and Mahoney et al. [20] which use the Barthel index scores, where low scores (≤60) indicate high dependency, intermediate scores (61–90) present a greater capacity to carry out activities, and high scores (90–100) tend to be more autonomous and functionally active.

The 5-STS test was used as a screening test since there are studies that relate sedentary lifestyle and physical activity levels with chair stand test performance [23,24,25]. The test has shown excellent reliability (ICC = 0.937) [26]. This test is normally used in nursing homes because of the limited space required as it can be easily performed. A standardization for carrying out the test was agreed upon with the therapists prior to the start of the study, with the patient’s arms crossed, a comfortable position in the chair, and a height of 42 cm from the floor [27]. Those older adults who take more than 15 s receive 1 day a week, those who take between 12 and 15 receive 2 days, and those who take <12 receive 3 days a week. This division was previously agreed upon by the therapists based on the criteria of the following literature [28] where they described that people who last 15 s or more are more prone to falls. However, a general agreement on this is missing in the current literature. The physical activity that the 3 groups received was carried out by the physiotherapists from the residences. The sessions consisted of guided joint mobility exercises at the beginning for 20 min (upper and lower extremities) and later psychomotor exercises combining upper trunk with lower extremities. The three groups carried out the same programs lasting 50 min.

A total of 76 older adults were recruited with a mean age of 84.29 ± 8.88 years. The average height was 1.53 ± 0.09 m, mean weight was 64.99 ± 12.93 kg, and mean BMI was 27.20 ± 4.83. From the 76 participants, 84% of the sample were woman (63 females and 13 males).

### 2.3. Settings

The measurements took place from December 2022 to December 2023. The evaluations were all carried out in the respective nursing homes by external evaluators with the supervision of the physiotherapist from the residences. The first assessment carried out in nursing homes was blood tests. These measurements were carried out by trained nurses from the Consorci Sanitari de Terrassa (CST). After obtaining the blood samples, subsequent measurements were carried out.

### 2.4. Assessments

The assessments can be grouped into demographic and anthropometric, muscular, functional, cognitive, and blood test parameters. Demographic and anthropometric variables included age (years), height (meters), weight (kg), body mass index (BMI), and gender (M/F). Muscular variables included handgrip strength, knee extension force (Newtons), and leg muscle power (Watt). Blood test variables included C-reactive protein (CRP) and ultra-sensitive C-reactive protein as inflammation markers. Functional variables included weekly exercise, the SPPB test, the 4-Meters Walking Test, and walking speed. Cognitive status was analyzed with the Mini Exam of Lobo (MEC).

#### 2.4.1. Demographic and Anthropometric Assessments

The residence physiotherapist provided age, height, and weight through data found in clinical history. The principal investigator calculated BMI using the formula BMI = kg/m^2^.

#### 2.4.2. Muscular Assessment

Handgrip strength was assessed with the Jamar hand dynamometer (Sammons Preston, Inc., Bolingbrook, IL, USA), in kilograms. Participants were seated with their elbows flexed at 90° and performed maximal handgrip strength for three seconds three times with each hand. The mean of the three attempts on each arm was used in the analysis. This procedure has been shown to have excellent reliability and validity (ICC = 0.85–0.98) [29]. The European Sarcopenia Study Group uses hand-grip cutoff values of <16 kg for women and <27 kg for men [30].

The knee extension force of the dominant lower limb was registered in Newtons. A traction dynamometer (PCE Ibérica S.L., Albacete, Spain) was used. This dynamometer has a 5% accuracy when measuring and is highly reliable (ICC = 0.91; 95% CI = 0.76–0.97) [31]. Participants were seated with their hips and knees flexed at a 90° angle, and a strap was secured on the distal and anterior part of the dominant leg. They were instructed to execute a knee extension, exerting maximal isometric force for five seconds. The test was repeated three times, and the mean value was used for the analysis.

The linear encoder (Vitruve System SL, Málaga, Spain) measured dominant leg muscle power. Muscular power was evaluated during the sit-to-stand movement, which was performed 5 times. Participants were seated with their hips and knees flexed at a 90° angle. The Velcro fastener was placed at the level of the participant’s pelvis and the encoder was placed on the floor. The average of all repetitions was calculated to obtain the results [32] The construct validity of linear encoder measurement of sit-to-stand power was shown at functional level and morphological level for older women [32].

#### 2.4.3. Blood Tests Assessments

CRP is a measure of systemic inflammation that results from the body’s immune response, which is triggered to protect against infection and illness [33]. The normal levels of CRP in older adults vary, but CRP levels below 1 mg/L are generally considered low-risk for inflammation-related diseases, while levels between 1 and 3 mg/L suggest moderate risk. Higher levels above 3 mg/L indicate an increased risk of inflammation-related diseases [33]. Ultra-sensitive C-reactive protein values were only analyzed if the CRP results were below 0.50 mg/L. C-reactive protein was obtained through blood tests performed in the residences. The samples were analyzed in the CST laboratory using a turbidimetric immunoassay method to determine the levels of CRP. The blood tests were the first evaluation that was carried out on the participants, prior to the rest of the measurements. Trained nurses from the Consorci Sanitari de Terrassa were in charge of carrying out the analyses. Participants were previously notified to come fasting on the day of the analysis. The test was carried out only once in the same residence with a duration of approximately 5 min.

#### 2.4.4. Functional Assessment

The frequency of weekly exercise was determined through the medical history they presented upon admission, the objectives of the family members, and the initial evaluation of the physiotherapist through joint mobility in the upper and lower extremities, 5-Sit To Stand (5-STS), and Barthel scale scores (0–100).

Short Physical Performance Battery (SPPB). This assessment battery comprises three tests: a balance assessment, a four-meter walking speed test and a five-repetition chair sit-to-stand task. Each test yields a numeric score ranging from 0 to 4, and these scores are aggregated to establish a composite score spanning 0 to 12. The test–retest reliability of the battery has demonstrated a favorable to outstanding range (Intraclass Correlation Coefficient–ICC: 0.83–0.92). Moreover, the inter-rater reliability among older adults admitted on an acute basis was exceptional (ICC: 0.91) [34]. A cut-off point of ≤10 points has been shown to be a strong predictor of the loss of ability to walk 400 m with a sensitivity of 0.69 and a specificity of 0.84. A cut-off point of ≤8 points has been shown to be associated with mobility-related disability and a very low SPPB test (0–6 points) has been shown to be associated with an increased risk of death [30].

Walking Speed. This is a functional test that reflects a subject’s average speed. Its reliability has been previously studied (ICC = 0.96, 95%CI = 0.94–0.98; SEM = 0.01). The score for this variable was obtained by dividing 4 between the results obtained from the 4 m walking test. The subjects had to walk 4 m as fast as possible. The test was performed twice and the average of the two tests was analyzed [35]. A cut-off of <0.8 m/s for a 4 m distance identifies subjects with poor physical performance [30].

#### 2.4.5. Cognitive Assessment

The evaluation of the cognitive status of the participants will be carried out through the Mini Exam of Lobo. The test shows a high validity for the evaluation of cognitive deterioration in the older population [36]. This questionnaire assesses the following cognitive aspects: temporal–spatial orientation, immediate and long-term memory, attention, calculation, language, abstract reasoning, and praxis. Scores vary between 0 and 35, with 0 being the minimum score and 35 being the maximum score. Before starting the test, the principal researcher explained the test and what its purpose was, together with the different aspects that make up the test. After that, the principal researcher administered the test individually to each participant, with an approximate duration of 10/15 min, and the total sum of all of the parts was used to calculate the test results. We considered using the Spanish version of the MMSE (MEC-35) to assess global cognition and to observe if there was any change in cognitive functions, because some items of the original version of Folstein are difficult for patients with a low cultural level, which affects the scale’s discriminative capacity.

#### 2.4.6. Statistical Analysis

The statistical analysis was conducted using SPSS version 26 (IBM, Armonk, NY, USA). For quantitative variables, the mean and standard deviation were reported, while percentages were used to describe qualitative or categorical variables. The Kolmogorov–Smirnov test was applied to assess whether the variables followed a normal distribution. Descriptive statistics were performed for all variables. Additionally, Pearson correlation analysis was used to examine relationships between all of the studied variables and physical activity levels. For those variables that present a significant correlation in the Pearson analysis, a multiple linear regression was subsequently performed to evaluate how physical activity levels affect these variables. To investigate whether the frequency of weekly physical exercise influenced the measured variables, a one-way ANOVA was performed. A significance level of (*p* < 0.05) was set, meaning that any *p*-value below this threshold indicates statistically significant differences between at least two of the groups. The effect size was calculated with Cohen’s d coefficient. Cohen’s coefficients were interpreted as follows: large effect sizes, d > 0.8; moderate effect sizes, d = 0.5–0.79; and small effect sizes, d = 0.2–0.49. If significant differences were detected through the ANOVA, a Bonferroni post hoc test was conducted to identify specifically which groups differed from one another. The Bonferroni method adjusts the significance threshold to account for multiple comparisons, reducing the risk of false positives. A (*p* < 0.05) was again used to determine statistical significance in these pairwise comparisons.

## 3. Results

**Figure 2:**
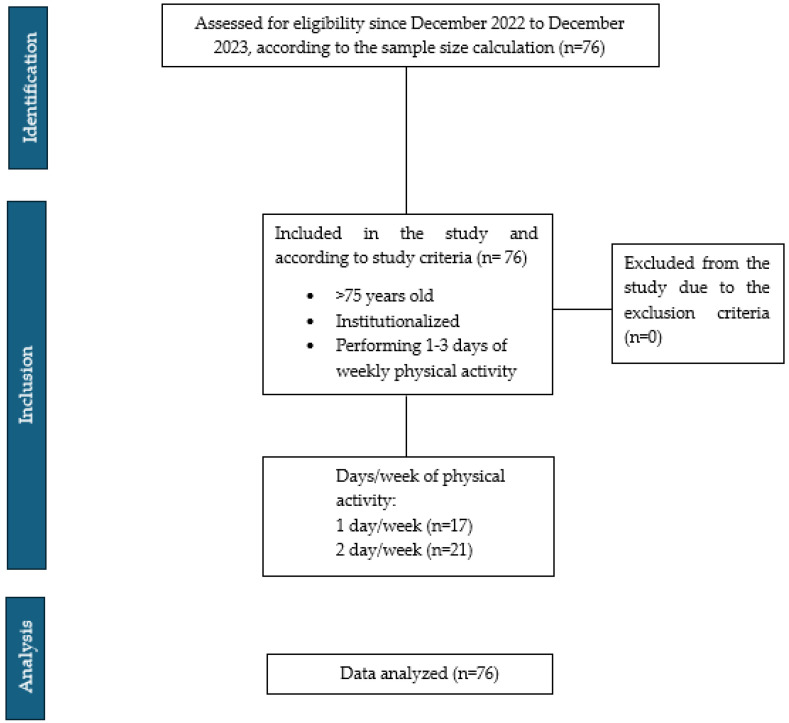
STROBE flow chart.

Between December 2022 and December 2023, 76 participants were recruited. Figure 2 shows the inclusion of participants according to the STROBE flow chart, with group assignments detailed in the same figure. The muscular, functional, and cognitive characteristics and inflammation markers are summarized in Table 1.

A Pearson correlation analysis (Table 2) revealed a moderate positive correlation between weekly physical exercise and MEC test scores (r = 0.53; *p* < 0.01), and a moderate negative correlation with CRP levels (r = −0.46; *p* < 0.01) and ultra-sensitive C-reactive protein (r = −0.33; *p* < 0.01). The regression model revealed that weekly physical activity levels could be a predictor for CRP levels (B = −0.491; R^2^ = 0.202; *p* < 0.001) and MEC punctuation (B = 4.52; R^2^ = 0.270; *p* < 0.001). A multiple linear regression analysis was not conducted for ultra-sensitive C-reactive protein due to severe multicollinearity with CRP.

**Figure 3:**
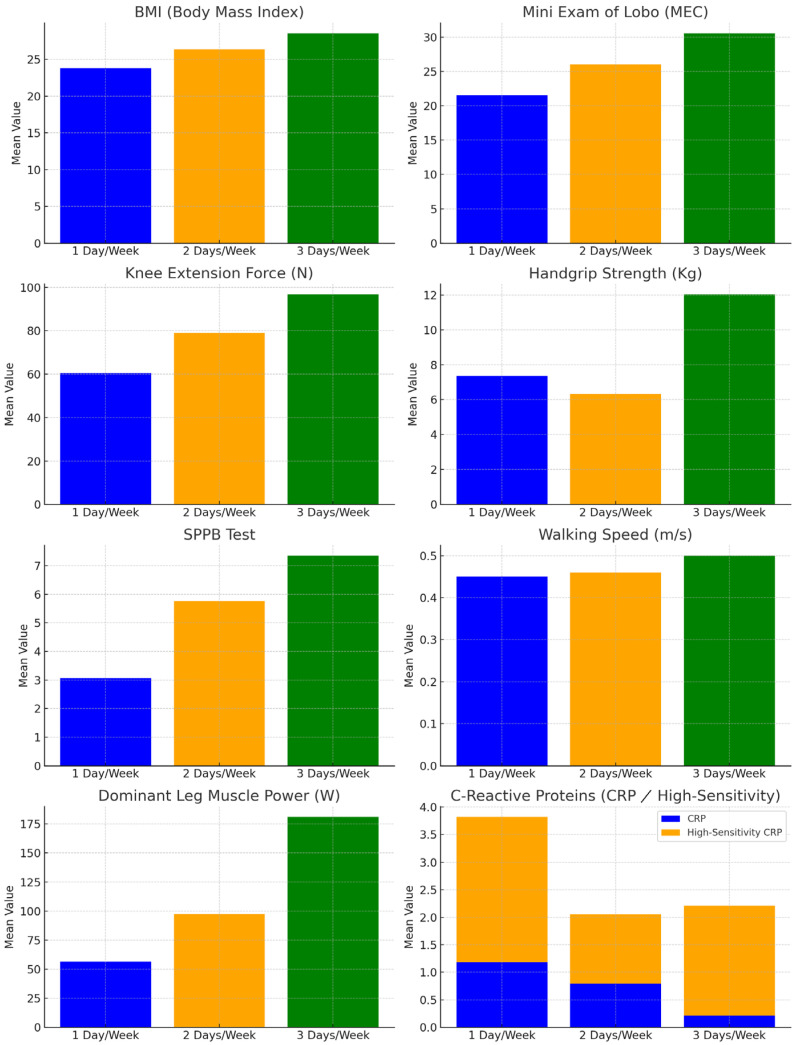
Differences between groups of weekly physical activity levels and the variables measured.

Participants were grouped by the number of PA days per week for a one-way ANOVA to assess differences in measurements among the three groups. The results are shown in Table 3 and Figure 3, for a better visual understanding of the differences.

### 3.1. Anthropometric Assessments

Significant differences were found in BMI (*p* < 0.01), with the one-day exercise group showing the lowest weight and BMI. Bonferroni post-hoc analysis revealed significant differences between the one-day and three-day exercise groups (*p* < 0.01). The post-hoc results are in Table 4.

### 3.2. Muscle Assessments

Significant differences were observed in the strength of the dominant lower extremity (*p* < 0.01), muscular power (*p* < 0.01), and hand grip strength (*p* < 0.01), with values of the group of one day/week being the lowest. Post-hoc analysis revealed a statistically significant difference (*p* < 0.02) for the handgrip strength between the group who did two days/week of PA and the group who did three days/week of PA. Statistically significant differences were found for the dominant leg strength (*p* < 0.01) and the muscle power (*p* < 0.01) between the group who did one day/week of PA and the group who did three days/week of PA.

### 3.3. Functional Assessment

Significant differences were observed for the SPPB test (*p* < 0.01) and for the walking speed (*p* < 0.01), with the values of the group that received one day/week being the lowest. Significant differences (*p* < 0.03) were found for the SPPB test between the group who did one day/week of PA and the group who did two days/week of PA, the group who did two days/week of PA and the group who did three days/week PA (*p* < 0.03), and between the group who did one day/week of PA and the group who did three days/week of PA (*p* < 0.01). For the walking speed, statistically significant differences (*p* < 0.02) were found between the group who did one day/week of PA and the group who did three days/week, and between the group who did two days/week and the group who did three days/week (*p* < 0.01).

### 3.4. Cognitive Assessment

Significant differences were observed for the MEC test (*p* < 0.01), with the values of the group that received one day/week of PA being the lowest. Statistically significant differences (*p* < 0.01) were found for the MEC test between the group who did one day/week of PA and the group who did three days/week, and between the group who did two days/week of PA and the group who did three days/week (*p* < 0.01).

### 3.5. Blood Assessment

Significant differences were found between groups for CRP (*p* < 0.01) and ultra-sensitive C-reactive protein (*p* < 0.02). Statistically significant differences were noted for CRP between the one-day and three-day groups (*p* < 0.01), and for ultra-sensitive C-reactive protein between the one-day and two-day groups (*p* < 0.02).

## 4. Discussion

### 4.1. Physical Activity as a Sarcopenia Stopper

The results of this study indicate that higher levels of weekly PA promote higher leg muscle strength and power, higher physical performance, and better cognitive function. Our findings resemble the following study [37] where they found also that higher levels of PA indicate greater muscle strength. Possible reasons for this are that exercise induces the production of adenosine triphosphate in skeletal muscle mitochondria, which improves aerobic capacity and promotes muscle protein synthesis, and affects the expression of muscle growth inhibitors and autophagy proteins mRNA (messenger RNA), which is an important strategy for preventing muscle atrophy and increasing muscle strength [38].

Added to all of this, we could understand that those participants with a higher level of weekly physical activity, and therefore greater muscle strength and cognitive function, also have greater physical performance, as previous studies have already indicated a relationship between the muscle strength of different muscles of the lower extremity and better physical performance, such as walking speed or getting up and sitting down from a chair [39]. Moreover, and related to muscle strength and physical performance, The European Working Group on Sarcopenia in Older People (EWGSOP2) defined that a sarcopenia diagnosis is confirmed by the presence of low muscle strength, and if we find also low muscle mass and low physical performance, sarcopenia can be considered severe [40]. This is relevant, because although we know that to define sarcopenia, muscle mass is measured by DXA, most of the time this is relatively costly, and it is not always easily and routinely used [41], while muscle strength and physical performance can be assessed clinically and easily in nursing homes, and we can have an approximation of the risk of sarcopenia in older adults.

Our findings have different clinical implications in terms of sarcopenia in institutionalized older adults. Abellan Van Kan G et al. [42] described a cut-off point of >0.8 m/s as a risk for sarcopenia. Cruz-Jentoft AJ [43] described an SPPB punctuation ≤ 8 as a cut-off point for the diagnosis of sarcopenia and low muscle strength as probable sarcopenia. Moreover, several studies [44,45] have related low BMI with a higher risk of sarcopenia, and one study [46] found that an underweight BMI was significantly associated with increased odds of sarcopenia. If we take into account all of this previous information, we can see in our results how those participants who only perform 1 day of physical activity per week could present a probable sarcopenia because of their lowest levels of handgrip, physical performance, walking speed, muscle power, and cognitive function. The results obtained, after being compared with previous information, allow us to see with caution that in the institutionalized study population, even if they are over 80 years old, exercising 3 days a week can reduce the probability of presenting sarcopenia and therefore, maintain healthy aging. Our results can also be transferred and generalized to other residences in Spain, since even having 84% of women in the study, the prevalence that has been observed in other studies [47,48] is 70% of women, indicating that our results could be useful to other nursing homes.

### 4.2. Physical Activity and Cognitive Function in Institutionalized Older Adults

On the other hand, the fact that in our study higher levels of physical activity are related to better cognitive function can be explained because muscle strength is positively correlated with whole brain volume, white matter volume, and gray matter volume in the right temporal pole and bilateral anterior medial ventral tegmentum [49]. Studies have found that older adult individuals engaged in regular physical activity with higher levels of physical activity and stronger muscle strength have a reduced risk of cognitive decline by 30 to 46% [50], so we can expect that higher levels of PA increase muscle strength and positively affect working memory and therefore, cognitive function.

There are multiple pathways explaining how PA exerts beneficial effects on neurocognitive health [51]. A recent systematic review [52] has shown that PA affects neurogenesis and neuronal survival in the hippocampus. Others [53] have shown that PA increases the expression of neurotrophic factors involved in learning and memory, and increases cerebral vascularization and blood flow, which might allow for increased nutrients and oxygenation.

### 4.3. The Relationship of Systemic Inflammation, Physical Activity, and Skeletal Muscle Degradation

A few previous reports using general population samples have indicated that higher levels of physical activity are cross-sectionally associated with lower levels of inflammation markers such as CRP [54,55]. In the present study, the group with lower levels of weekly physical activity had the highest levels of CRP and the worse knee extension force and physical performance. A substantial body of literature has demonstrated that inflammatory cytokines activate many of the molecular pathways involved in skeletal muscle wasting leading to an imbalance between protein synthesis and catabolism, being negatively related to muscle strength and mass [56]. Moreover, the following study found that sarcopenia was associated with higher serum CRP levels, regarding the relationship between frailty and inflammation [57]. Physical activity can slow age-related changes in cytokine production [58]. Previous research results show that participating in physical activity lowers pro-inflammatory biomarkers, suggesting that older adults who participate in a regular exercise regimen will have less adipose tissue resulting in decreased production of pro-inflammatory and increased anti-inflammatory biomarkers and better physical function [58].

These previously described associations have important clinical implications in our study population, highlighting the importance of increasing physical activity levels [59] in the institutionalized elderly population to reduce the risk of sarcopenia and improve functionality and independence.

### 4.4. Need to Improve Adherence to Physical Activity in Institutionalized Older Adults

Even if they believe in the positive potential that physical activity has, there are several studies [7,60,61] that show that most middle-aged and older adults do not engage in the recommended amount of physical activity, and interventions designed to improve physical activity levels tend to have poor adherence. This can be explained, among other reasons, by aging expectations and sedentary habits, in addition to considering that they may have physical and cognitive limitations, especially during institutionalization [7,62]. It is of vital importance, and even more so considering that we are dealing with a specific vulnerable population, that we find new therapeutic strategies that can not only improve health parameters, reducing sarcopenia and frailty, but that these new therapeutic tools generate adherence to physical activity so that it can become a regular daily activity. In this sense, Yang Y et al., found in their study that environmental context and resources, social influences, skills, reinforcement, and emotions were identified as the five theoretical domain framework domains that have the greatest impact on exercise adherence among older adults [63]. Therefore, as seen before by Gomez-Redondo et al., applying motivational strategies might help increase adherence to exercise programs, which could eventually result in greater training-induced adaptations [64]. Another factor to take into account to obtain greater training-induced adaptations, may be the use of nutritional supplementation, since in a recent meta-analysis [65] it was observed that supplementation such as protein, creatine, or leucine with different types of training exercises can help older people with sarcopenia to improve muscle mass and muscle strength.

In summary, in a population where from 2050 onwards 20% will be over 60 years old [13], it is important that the core elements of national and international health policies include ensuring that older adults remain healthy and independent for as long as possible.

One of the limitations is that the groups presented a very heterogeneous distribution in terms of number, and this could influence our results in favor of the 3 day/week group. The distribution made by therapists for the designation of weekly activity may be biased by the performance and interpretation of screening tests. Second, our study was based on observational data. Therefore, our findings may have been due to residual confounding from some of our control variables. In addition, there may have been some unknown confounders that we did not control for, such as the level of physical activity previous to the institutionalization, and these unknown confounders may have affected our results. It would be very interesting in the future to have relevant data on participants such as the date of entry into the residence, levels of physical activity throughout their lives, and previous pathologies, to better understand the health parameters that are evaluated. Another limitation of this study is the extrapolation of the multiple linear regression results based on our observational study, which prevents establishing causal relationships and limits the ability to extrapolate the results of the linear regression over time. A longitudinal study and with non-institutionalized older adults, would be necessary to analyze how these variables change over time and determine potential causal effects.

## 5. Conclusions

This study highlights that institutionalized older adults engaging in PA three days per week demonstrate greater physical performance, greater muscle strength and power, and less cognitive decline. Improving adherence to regular physical activity in older adults, and especially in institutionalized people, can be an important factor in improving their health parameters. For this reason, it is necessary to implement regular weekly physical activity programs in nursing homes that motivate and encourage adherence to exercise among institutionalized older adults. This way, this specific population can reduce their risk of sarcopenia and achieve the healthiest aging possible.

## Figures and Tables

**Table 1:** Characteristics of the participants.

Variables	Mean ± SD (N = 76)
Age (years)	84.2 ± 8.8
Sex (n%) Men Women	13 (16%) 63 (84%)
Height (meters)	1.5 ± 0.0
Weight (kg)	64.9 ± 12.9
BMI	27.2 ± 4.8
Technical aids (%)	10.52%
Barthel Index	82.15 ± 18.4
Weekly Exercise 1 day/week 2 day/week 3 day/week	17 (22%) 21 (27%) 38 (51%)
Mini Exam of Lobo (MEC)	27 ± 7
Knee extension force (Newtons)	81.6 ± 42.5
Handgrip strength (kg)	8.7 ± 7.7
SPPB Test	5.9 ± 3.6
Walking speed (m/s)	0.6 ± 0.4
Dominant leg muscle power (Watt)	144.9 ± 110.6
C-reactive protein (CRP) (mg/L)	0.4 ± 0.6
Ultra-sensitive C-reactive protein (mg/L)	1.8 ± 1

Abbreviations: BMI (Body Mass Index); kg (kilograms); SD (Standard Deviation); MEC (Mini-Exam of Lobo); SPPB (Short Physical Performance Battery); C-reactive protein (CRP); m/s (meters per second).

**Table 2:** Correlations between weekly physical activity levels and health parameters.

Weekly Exercise		
Variables	Rho	*p*-Value
Age (years)	0.16	0.17
Height (meters)	−0.02	0.85
Weight (kg)	−0.06	0.64
BMI	−0.21	0.13
Mini Exam of Lobo (MEC)	0.53	0.00
Knee extension force (Newtons)	−0.08	0.46
Handgrip strength (kg)	0.04	0.67
SPPB test	0.03	0.75
Walking speed (m/s)	−0.18	0.12
Dominant leg muscle power (Watt)	0.05	0.63
Glycated hemoglobin (HbA1c)	0.03	0.85
C-reactive protein (CRP)	−0.46	0.00
Ultra-sensitive C-reactive protein	−0.33	0.00

Abbreviations: BMI (body mass index); kg (kilograms); SD (standard deviation); MEC (Mini-Exam of Lobo); SPPB (Short Physical Performance Battery); CRP (C-reactive protein); m/s (meters per second).

**Table 3:** Differences between groups of weekly physical activity levels.

	1 Day/Week (n = 17)	2 Day/Week (n = 21)	3 Day/Week (n = 38)		
Variables	Mean ± SD	Mean ± SD	Mean ± SD	*p*-Value	Effect Size
Age (years)	86.65 ± 7.75	87.00 ± 7.31	81.27 ± 9.82	0.06	0.69
BMI	23.79 ± 2.72	26.36 ± 3.71	28.56 ± 4.57	0.00	0.96
Mini Exam of Lobo (MEC)	21.52 ± 7.11	26.01 ± 6.61	30.58 ± 5.10	0.00	1.21
Knee extension force (Newtons)	60.52 ± 31.11	79.00 ± 39.98	96.74 ± 45.91	0.01	0.70
Handgrip strength (kg)	7.35 ± 8.22	6.32 ± 5.64	12.03 ± 8.05	0.01	0.45
SPPB test	3.06 ± 3.51	5.76 ± 2.53	7.95 ± 3.27	0.00	1.46
Walking speed (m/s)	0.45 ± 0.37	0.46 ± 0.20	0.81 ± 0.50	0.00	0.87
Dominant leg muscle power (Watt)	56.60 ± 97.94	127.45 ± 58.94	190.86 ± 117.49	0.00	1.09
C-reactive protein (CRP)	1.18 ± 0.36	0.79 ± 0.88	0.21 ± 0.17	0.00	1.73
Ultra-sensitive C-reactive protein	2.64 ± 1.08	1.26 ± 0.50	2.00 ± 0.87	0.02	1.18

Abbreviations: BMI (body mass index); MEC (Mini-Exam of Lobo); SD (standard deviation); CRP (C-reactive protein); SPPB (Short Physical Performance Battery).

**Table 4:** Differences in means between the three weekly physical activity groups and the level of statistical significance.

	Difference 1 Day/Week–2 Day/Week			Difference 1 Day/Week–3 Day/Week			Difference 2 Day/Week–3 Day/Week		
Variables	Mean Difference (95%CI)	*p*-Value	Effect Size	Mean Difference (95%CI)	*p*-Value	Effect Size	Mean Difference (95%CI)	*p*-Value	Effect Size
BMI	−2.57 (−6.61; 1.46)	0.36	0.76	−4.77 (−8.47; −1.08)	0.00	1.16	−2.21 (−5.34; 0.93)	0.26	0.50
Mini Exam of Lobo (MEC)	−4.57 (−9.54; 0.38)	0.08	0.65	−9.05 (−13.47; −4,64)	0.00	1.56	−4.48 (−8.74; −0.22)	0.00	0.81
Knee extension force (Newtons)	−18.48 (−52.01; 15.05)	0.54	0.51	−36.21 (−65.88; −6.55)	0.01	0.86	−17.74 (−45.81; 10.34)	0.37	0.40
Handgrip strength (kg)	1.04 (−4.96; 7.03)	1.00	0.14	−4.67 (−10.04; 0.69)	0.10	0.57	−5.70 (−10.71; −0.70)	0.02	0.78
SPPB test	−2.70 (−5.21; −0.19)	0.03	0.89	−4.88 (−7.14; −2.63)	0.00	1.46	−2.18 (−4.28; −0.08)	0.03	0.72
Walking speed (m/s)	0.00 (−0.36; 0.36)	1.00	0.03	−0.36 (−0.69; −0.03)	0.02	0.77	−0.36 (−0.64; −0.07)	0.00	0.83
Dominant leg muscle power (Watt)	−70.85 (−151.79; 10.09)	0.10	0.90	−134.26 (−209.15; −60.37)	0.00	1.20	−63.41 (−129.50; 2.68)	0.06	0.62
C-reactive protein (CRP)	0.38 (1.27; −0.50)	0.85	0.55	0.97 (1.66; 0.27)	0.00	3.90	0.58 (1.39; −0.22)	0.23	1.07
Ultra-sensitive C-reactive protein	1.38 (0.142; 2.62)	0.02	1.70	0.64 (−0.39; 1.67)	0.37	0.68	−0.74 (−1.82; 0.34)	0.27	0.97

Abbreviations: BMI (body mass index); kg (kilograms); MEC (Mini Exam of Lobo); CRP (C-reactive protein).

## Data Availability

Data are available from the first author upon reasonable request and with the permission of the Consorci Sanitari De Terrassa. Please contact the first author for data.

## Abbreviations

The following abbreviations are used in this manuscript:

BMI	body mass index
kg	kilograms
SD	standard deviation
MEC	Mini-Exam of Lobo
SPPB	Short Physical Performance Battery
CRP	C-reactive protein
M/s	Meters per second
